# Medical Students and Childhood Obesity: Health Disparity and Implication for Education

**DOI:** 10.3390/ijerph16142578

**Published:** 2019-07-19

**Authors:** Shinduk Lee, Matthew Lee Smith, Laura Kromann, Marcia G. Ory

**Affiliations:** 1 Center for Population Health and Aging, Texas A&M University, College Station, TX 77843, USA; 2 Department of Environmental and Occupational Health, Texas A&M University, College Station, TX 77843, USA; 3 Department of Health Promotion and Behavior, University of Georgia, Athens, GA 30605, USA; 4 Texas A&M College of Medicine, Round Rock, TX 78665, USA

**Keywords:** childhood obesity, health disparity, medical training, medical students’ perception, stigma

## Abstract

This study aimed at examining the relationship between medical students’ perceptions about health disparity and childhood obesity care. A cross-sectional survey (*n* = 163) was used to examine medical students’ characteristics and perceptions related to health disparity and childhood obesity. Multiple mixed effects regression models with Tukey’s tests were used to examine participants’ perceived importance of different roles (e.g., parents) and topics to discuss with child patients and their parents. Separate models were used to examine whether health disparity perception was associated with participants’ perceived importance of different roles and topics to discuss with child patients and their parents. Despite acknowledging that low-income families might lack resources to change health behaviors, many medical students still reported patients and parents being primarily responsible for childhood obesity condition. Participants perceived that the most important topic to discuss was patient’s behaviors, followed by access to safe environments and school-based interventions. Participants’ perception about health disparity was significantly associated with their perceived importance of different roles and topics to discuss with parents. The current study implies disconnection in linking health disparity with childhood obesity among medical students and confirms the importance of sensitizing medical students about the socio-environmental determinants of childhood obesity.

## 1. Introduction

About one-in-five American children are affected by childhood obesity [1], a strong predictor of avoidable obesity-related health conditions and health care costs throughout the life-course [2]. Childhood obesity is a complex problem that results from interacting social, behavioral, environmental, biological, and genetic factors [2,3]. While childhood obesity can occur in any socio-economic or ethnic group, Hispanic and non-Hispanic black individuals [1,4,5] and those with lower socioeconomic backgrounds [6] are at increased risk for obesity. In considering solutions, the influence of healthcare professionals in childhood obesity care cannot be overlooked as physicians play important roles in influencing patient behavior [7,8,9].

Universal evaluation that integrates annual body mass index (BMI) examination with other clinical assessments can help with diagnosing childhood obesity, identifying the etiology, and providing necessary support for primary and secondary prevention [2,3]. Several primary-care based childhood obesity interventions have been shown to be feasible and effective in addressing childhood obesity [10,11]. Primary-care-based interventions can be particularly effective among patients from ethic minority or lower socioeconomic backgrounds [10,12], providing potential benefits to reduce disparity among childhood obesity.

Literature about childhood obesity perceptions by medical students suggest that medical students feel unprepared to address childhood obesity and additional training is needed. For example, using a nationally-representative sample from the United States (US), a study reported that less than 50% of graduating pediatric residents received the comprehensive training in childhood obesity care, with the least trained in behavioral management strategies and childhood obesity advocacy [13]. Another study suggested medical school curriculum should include more observations and practices for medical students to enhance students’ understanding of the childhood obesity epidemic and their roles in childhood obesity care [14]. Unfortunately, there is a lack of studies to inform the medical school curriculum development, especially from the point of view of students themselves. Furthermore, while there is much literature about disparity in childhood obesity prevalence and care, there is a gap in the knowledge about the association between medical students’ perspectives about health disparity and their perceptions towards childhood obesity care.

Therefore, this study aimed at examining medical students’ perceptions related to health disparity and childhood obesity. We examined: (1) perceived importance of different roles in childhood obesity care, (2) perceived importance of different topics to be discussed with child patients and their parents, and (3) associations between medical students’ perspectives about health disparity and their perceived importance of different roles and discussion topics in childhood obesity care.

## 2. Materials and Methods

### 2.1. Study Participants

The study participants were first to fourth year medical students from a public medical school in Central Texas. Using the medical school listserv, the email invitation to participate in an anonymous online survey was sent to the medical students. The email invitation was sent to all medical students during March–April 2012, and 163 (about 20%) returned the survey. Consent to participate in the study was embedded in the first page of the online survey, with only those consenting permitted survey access. IRB approval was obtained through Texas A&M University (Protocal number: 2011-0905).

### 2.2. Data Collection

A cross-sectional survey instrument was used to examine medical students’ demographics (e.g., age, sex, and race), year in medical school, desired fields of medicine, preference to work with patients younger than 18 years old, and perceptions related to childhood obesity care.

#### 2.2.1. Perceived Importance of Different Roles in Reducing Childhood Obesity

Participants were asked to report how important each of the following roles in reducing childhood obesity: parent, patient, physician, teacher, friend, sport coach, minister, municipal politician (e.g., mayor), and state-level politician (e.g., governor). The answer ranged from ‘not at all important’ (= 1) to ‘very important’ (= 5). Participants’ rated importance of patient, parent, and friends were averaged, and the composite score was named ‘patient and informal roles.’ Similarly, participants’ rated importance of teacher, sport coach, and minister were averaged, and the composite score was named ‘formal roles.’ Participants’ rated importance of physician was considered individually. Participants’ rated importance of municipal politician and state-level politician were averaged, and the composite score was named ‘government.’

#### 2.2.2. Perceived Importance of Different Discussion Topics

Participants were also asked to rate importance of discussing different topics related to childhood obesity with child patients and patients’ parents: “In treating childhood obesity, how important are each of the following to discuss with parents/children?” The topics asked included: limiting sugar-sweetened beverages, limiting energy-dense/nutrient-poor foods, cutting back on fast food, age-appropriate portion sizes, limiting screen time, increasing physical activity, healthy body image, adequate intake of fruits, vegetables, calcium, and fiber, having access to safe environments for being physically-active, and having mandated health promotion programs in schools. Each item was rated from ‘not at all important’ (= 1) to ‘very important’ (= 5). All the patients’ or parents’ behavior-related items (e.g., limiting sugar-sweetened beverages, limiting energy-dense/nutrient-poor foods, cutting back on fast food, age-appropriate portion sizes, limiting screen time, increasing physical activity, healthy body image, adequate intake of fruits, vegetables, and calcium, and fiber) were averaged into one composite score.

#### 2.2.3. Perceptions Related to Childhood Obesity

Multiple single-item measures were used to examine participants’ perceptions related to childhood obesity. Each item was dichotomized into interpretable categories to address skewness and bimodality of the response distribution. Perceived significance of childhood obesity as a health problem was examined using the two questions: “Today, how likely is it that the physician will currently encounter (obesity) during a routine medical visit with children?” and “Please indicate if you believe (obesity) will affect more or less of the adolescent population a decade from now.” The first question used 7-point Likert scale ranging from ‘very unlikely’ to ‘very likely’ and was dichotomized into ‘likely’ (= 1) and ‘neutral or unlikely’ (= 0). The second question used 3-point Likert scales, and was dichotomized to ‘more of the population’ (= 1) and ‘same or less of the population’ (= 0).

The online survey included multiple 5-point Likert items about the extent of agreement with the following statements: (1) “childhood obesity is a family matter,” (2) “parents are primarily responsible for their child’s weight,” and (3) “physicians are not trained to deal with childhood obesity.” The valid responses ranged from ‘strongly disagree’ to ‘strongly agree’ and were dichotomized into ‘agree’ (= 1) and ‘neutral or disagree’ (= 0). On 5-point Likert items, participants were also asked to rate physicians’ and participants’ effectiveness in treating childhood obesity, and the ratings were dichotomized into ‘effective’ (= 1) and ‘neutral or ineffective’ (= 0). Similarly, on a 5-point Likert item, participants’ preparedness to treat childhood obesity was rated from ‘not at all prepared’ to ‘very prepared’ and was dichotomized into ‘prepared’ (= 1) and ‘neutral or not prepared’ (= 0).

#### 2.2.4. Health Disparity

Participants were asked to rate how much they agreed with the following statements: “Low-income families don’t have the knowledge to change their current health behaviors,” and “Low-income families don’t have the monetary means to change their health behaviors.” Each item was measured using a 5-point Likert item, and ranged from ‘strongly disagree’ (= 1) to ‘strongly agree’ (= 5). A composite scale was calculated by averaging the two items. Similarly, a 5-point Likert item was used to rate each participant’s level of agreement with the following statement: “The average American does not have the resources to change their health behaviors.”

### 2.3. Statistical Analysis

First, the characteristics and perceptions of study participants were described using frequency and percentage for categorical variables and means and a 95% confidence interval (CI) for interval variables. Participants’ responses to the health disparity score was dichotomized into low (not agreeing that low-income families lack resources to change their health behaviors) or high (agreeing that low-income families lack resources to change their health behaviors) awareness of health disparity. Then, the relationship between participants’ health disparity perception and participants’ characteristics and other perceptions were examined using bivariate analyses. Bivariate analyses were performed using both raw and dichotomized (median split) health disparity scores. Since there were no meaningful differences in the statistical significance, we reported the results from the bivariate analyses using the dichotomized health disparity measure (Chi-square test for categorical variables and ANOVA for interval variables). The dichotomized health disparity score was only used for the bivariate analyses. In addition, pairwise t-test was used to compare participants’ perceptions about the sufficiency of resources to change health behaviors among low-income American families and average Americans.

Multiple mixed effects regression with Tukey’s post-hoc tests were used to compare participants’ perceived importance of different roles in reducing childhood obesity, as well as perceived importance of different topics to discuss with child patients and patients’ parents. Fixed effects were ‘roles’ for the first model and ‘topics’ for the second and third models. Furthermore, three additional mixed effect regression models were performed to examine whether health disparity perception was associated with differences in perceived importance of roles and discussion topics. Appropriate interaction terms (e.g., roles x health disparity perception, topic for parents x health disparity perception, or topics for children x health disparity perception) were used in each additional model. For all models, participant-level random effects were used to account for individual-level variation in their rating of perceived importance. All models were adjusted for the variables that were significantly different between participants with low and high awareness of health disparity based on the bivariate analyses (e.g., age, race, and perception of physicians’ training in childhood obesity).

Missing response rates increased towards the end of the survey. The questions about sociodemographic characteristics were placed at the end of the survey, and the item about race had the highest missing response rate (*n* = 37, 23%). Participants who did not report race were less likely to report wanting to work with populations under 18 years old (16% versus 33%, *p* = 0.045). Listwise deletion was used for handling missing data. All statistical analyses were performed in 2018 using SAS software, version 9.4.

## 3. Results

### 3.1. Participant Characteristics

Table 1 presents sample characteristics by participants’ health disparity perception. On average, participants were about 25.6 years old (range: 22–41), and about 57% were female and 56% were White. Almost half of the participants indicated that they would like to practice specialty care, and about 30% reported that they would like to work with patients under 18 years old. Compared to participants with low awareness of health disparity, participants with high awareness of health disparity were younger (25.0 versus 26.3 years old, *p* = 0.047) and less likely to be White (45% versus 70%, *p* = 0.012).

### 3.2. Perceptions Related to Childhood Obesity

Table 2 shows participants’ perceptions related to childhood obesity. The majority viewed childhood obesity as a significant public health issue, reporting that it would be very likely to encounter childhood obesity in routine medical visits (95%) and that childhood obesity would affect greater numbers in the future (91%). About 68% perceived childhood obesity as a family matter, and over 90% reported parents being primarily responsible for their children’s weights. While about 35% reported that physicians were not trained to deal with childhood obesity, 70% reported that physicians can be effective in treating childhood obesity. On the other hand, less than 50% of participants reported that they were prepared to treat childhood obesity or would be effective in treating childhood obesity as a physician.

Participants indicated that compared to average Americans, low-income American families were more likely to lack resources to change health behaviors (3.45 versus 2.09, t = 16.42, df = 128, *p* < 0.001). Compared to participants with low awareness of health disparity, participants with high awareness of health disparity were less likely to agree that physicians were not trained to deal with childhood obesity (26% versus 48%, *p* = 0.008).

### 3.3. Perceived Importance of Different Roles in Reducing Childhood Obesity

**Figure 1:**
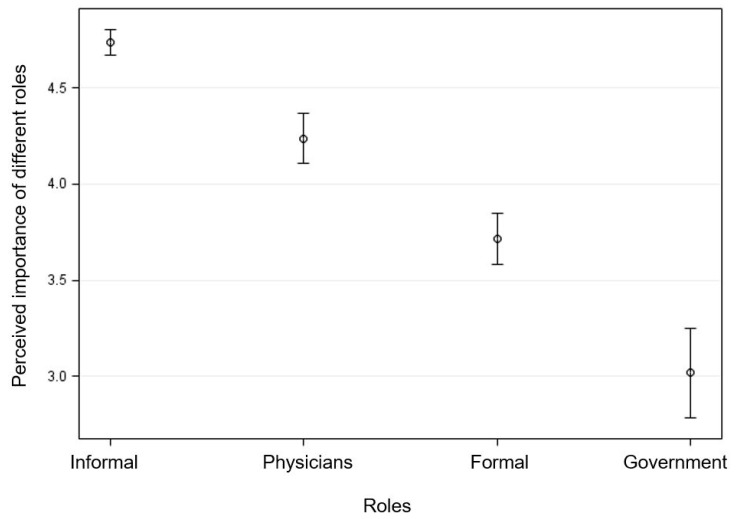
Estimated mean with 95% confidence interval for medical students’ perceived importance of different roles in reducing childhood obesity (score ranging from 1 to 5, with a higher score indicating greater importance).

After adjusting for participants’ age, race, and perception about physicians’ training in childhood obesity, participants’ perceived importance of different roles in reducing childhood obesity were significantly different across roles (F_3,106_ = 127.51, *p* < 0.001) (Figure 1). In addition, post-hoc Tukey comparisons showed that perceived importance of each role was significantly different from other roles (*p* < 0.05). The adjusted importance was highest for patients and informal roles (4.74, 95% CI = (4.67, 4.80)), followed by physicians (4.24, 95% CI = (4.11, 4.37)), formal roles (3.71, 95% CI = (3.58, 3.85)), and government (3.02, 95% CI = (2.78, 3.26)).

### 3.4. Perceived Importance of Discussing Different Topics with Child Patients and Their Parents

**Figure 2:**
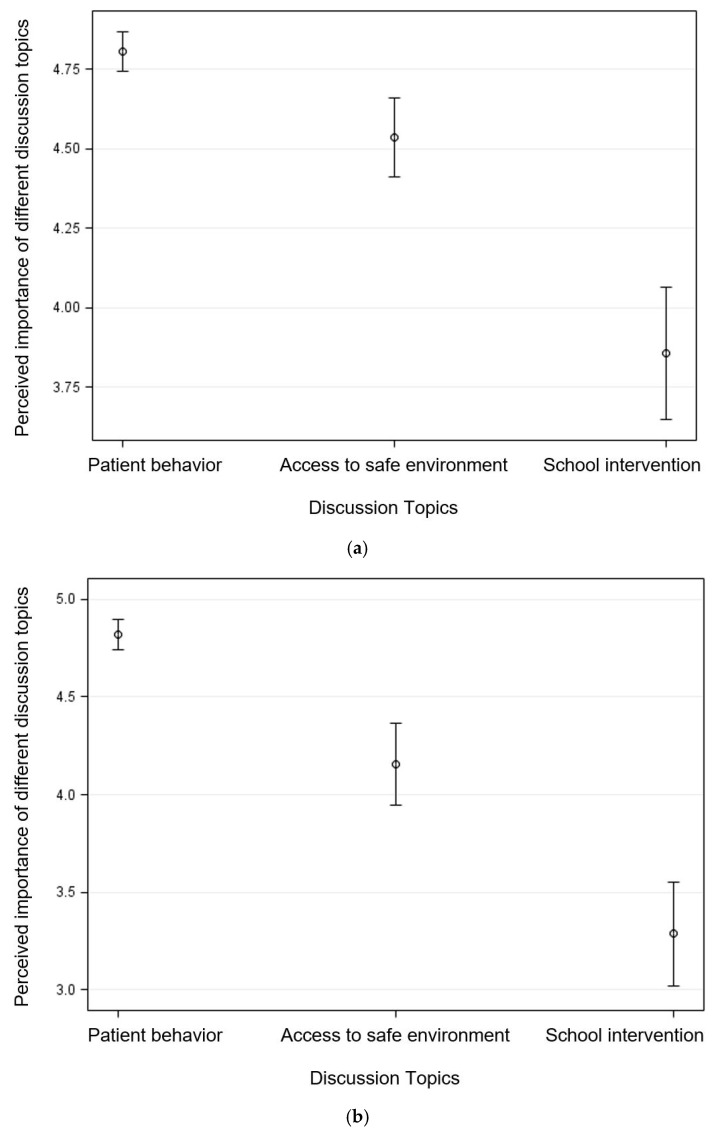
Estimated mean with 95% confidence interval for medical students’ perceived importance of different topics that can be discussed with (**a**) parents and (**b**) child patients (score ranging from 1 to 5, with a higher score indicating greater importance).

Participants’ perceived importance of discussing different topics with parents (F_2,107_ = 44.15, *p* < 0.001) and children (F_2,107_ = 71.29, *p* < 0.001) significantly varied by topics, after adjusting for participants’ age, race, and perceptions related to physicians’ training in childhood obesity (Figure 2). According to the Tukey’s post-hoc comparison, perceived importance was significantly different for each discussion topic (*p* < 0.05). With parents, participants perceived that it would be most important to discuss patients’ behaviors (4.81, 95% CI = (4.75, 4.87)), followed by access to safe environments (4.54, 95% CI = (4.41, 4.66)), and having mandated school interventions (3.86, 95% CI = (3.65, 4.07)). Similarly, participants perceived that it would be most important to discuss with child patients about patients’ behaviors (4.82, 95% CI = (4.74, 4.90)), followed by access to safe environments (4.16, 95% CI = (3.95, 4.37)), and having mandated school interventions (3.29, 95% CI = (3.02, 3.55)).

### 3.5. Association with Health Disparity Perception

**Figure 3:**
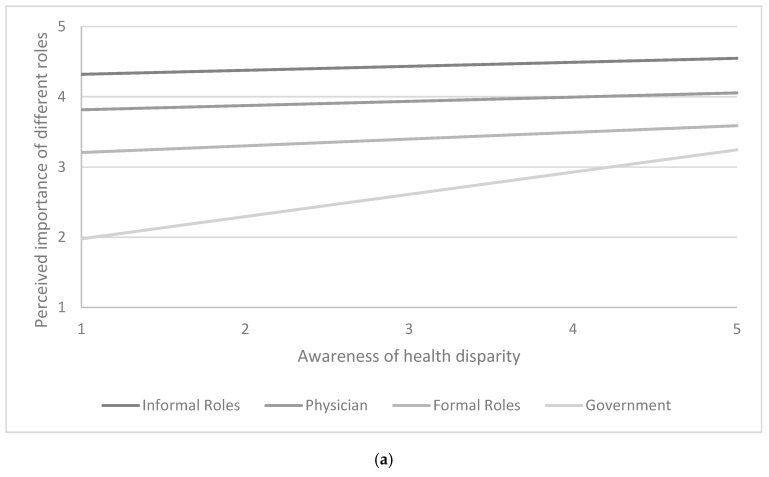

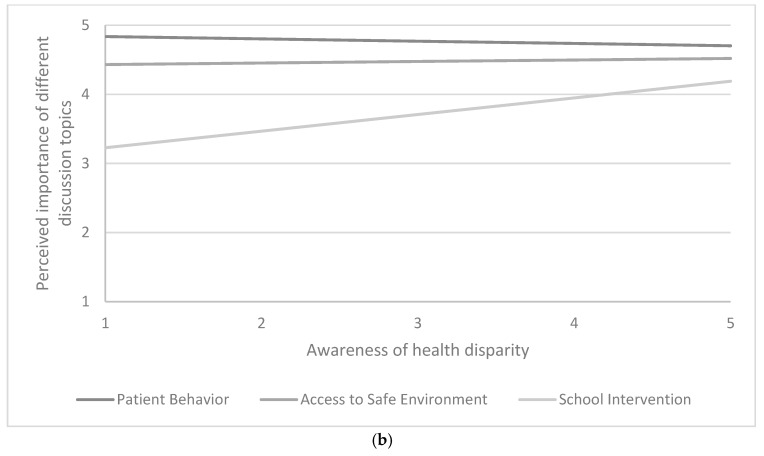
Estimated mean with 95% confidence interval for medical students’ perceived importance of (**a**) different roles, and (**b**) different topics that can be discussed with parents (scores ranging from 1 to 5, with a higher score indicating greater importance).

Participants’ perception that low-income families lack resources to change their health behaviors was significantly associated with participants’ perceptions about the importance of different roles and discussion topics (Table 3). While the overall F-statistics for the interaction terms were not statistically significant (*p* > 0.05), increased awareness of health disparity was positively associated with perceived importance of government roles (unstandardized regression coefficients (B) = 0.257, 95% CI = (0.003, 0.511), *p* = 0.048) and perceived importance of discussing with parents about having school-based health promotion interventions (B = 0.274, 95% CI = (0.053, 0.495), *p* = 0.016) (Figure 3). All regression models were performed after adjusting for participants’ age, race, and perceptions about physicians’ training in childhood obesity.

## 4. Discussion

The findings from the current study leverage the understanding of medical students’ perceptions regarding childhood obesity care. Participating medical students generally perceived childhood obesity as a prevalent health condition among child patients. Similarly, previous studies also reported that medical students consider childhood obesity as a prevalent health problem [15,16]. While medical students perceived physicians as the second most important role in addressing childhood obesity, less than half reported feeling prepared to address the health condition. About one-in-three reported that physicians are not trained to deal with childhood obesity. The current study re-emphasizes previous reports that medical students lack training related to childhood obesity care [13,14,17,18]. These observations are not surprising given the past reports about lack of comprehensiveness about childhood obesity care in medical school curricula [13,14,18] or the medical licensing examinations [19].

Among medical students, childhood obesity was largely considered as patients’ and parents’ responsibility, which might be related to victim-blaming. From the current study, medical students rated patient, parents, and friends as the most important roles in addressing childhood obesity issues, and government roles (e.g., politicians) as the least important. In addition, medical students reported patient behaviors as the most important topic to be discussed with child patients and patients’ parents, followed by access to safe environments and having school interventions. Blaming patients with obesity has been observed among various health professional students [16,20,21]. Other work has reported that about one-in-three medical students think that patients with obesity are not receptive to weight-loss recommendations and do not have the motivation to change their lifestyle [22]. Training for medical students should increase their understanding that “the deceptively simple issue of encouraging physical activity and modifying dietary habits, in reality, raises complex social and economic questions about the need to reshape public policy in food production, food manufacturing, healthcare, retail education, culture, and trade” [23].

Most importantly, the study highlights the need for sensitizing medical students regarding social determinants of health issues and approaches that incorporate these determinants. Medical students who recognized health disparity issues reported greater importance of the government’s role in reducing childhood obesity and discussing with parents about having school-based interventions. This study is one of the first studies to look at the relationship between medical students’ health disparity perception and their perceptions related to childhood obesity care, and the study findings are in line with findings from other related studies. For example, medical students from different racial and socioeconomic backgrounds indicated significantly different magnitudes of bias towards patients with obesity [24]. Medical students from different racial and socioeconomic backgrounds are likely to have different perceptions about health disparity, which is then associated with magnitudes of weight bias. Educating health professional students about the complex etiology of obesity and providing hands-on experience in community settings has been recommended to reduce weight bias and improve students’ attitudes towards patients with obesity [15,16,22].

Given the complexity of childhood obesity care, team approaches involving patients, family, friends, and professionals from various disciplines, as well as community and system are recommended to address the health issue [25,26]. While only limited studies exist, collaboration with community and interprofessional education have been shown to enhance medical students’ understanding of their roles and responsibilities in childhood obesity care [15,16]. For example, the Junior Doctors of Health© Interprofessional Education (JDOH IPE) program was designed to prepare students from various health science fields (e.g., medicine, pharmacy, social work, and public health) to address childhood obesity in low-income communities [16]. The JDOH IPE provided students hands-on experiences and didactic sessions about social determinants of childhood obesity, working in community settings, and roles of different healthcare professionals in childhood obesity care [16]. After participating, the JDOH IPE students reported significantly higher perceived importance of social and environmental determinants of childhood obesity and indicated better understanding of their professional roles and responsibility for childhood obesity care [16]. Larger, long-term evaluation of the interprofessional education program that involve necessary procedures to control for bias are needed to better inform medical educators [27].

### Limitations

Generalizability of the study findings is limited by using a small, non-random sample from a sample pool that might not be representative of a broader population (e.g., all US medical students). Furthermore, response rates might have been lower for medical students who would not want to work with a pediatric population, in comparison with those who would like to work with a pediatric population. However, no other difference was observed between participants who completed and did not complete the survey. While the study findings should be interpreted with caution, the study findings are well-aligned with the existing literature and make a unique contribution by exploring the relationship between medical students’ perceptions about health disparity and childhood obesity care.

## 5. Conclusions

Consistent with previous studies, this study shows that while medical students considered childhood obesity as a significant health problem influencing many children, medical students did not feel prepared for childhood obesity care. Despite acknowledging that low-income families might lack resources to change health behaviors, many medical students still reported patients and parents being primarily responsible for childhood obesity condition. This may imply disconnection in linking health disparity with childhood obesity care. The current study confirms the need for updating the medical school curricula to prepare medical students to address childhood obesity effectively, in and out of office settings. Not just in terms of knowledge, the updated medical education should be more comprehensive, sensitizing medical students about the complex etiology of childhood obesity and preparing them to work in teams with patients, parents, community, and professionals from other disciplines.

## Figures and Tables

**Table 1:** Participants’ characteristics by their perception about health disparity.

Variables	Mean (95% CI ^1^) or Frequency (%)			*p*-Value ^2^
	Overall (*n* = 163)	Low Awareness of Health Disparity (*n* = 53)	High Awareness of Health Disparity (*n* = 78)	
Age (years)	25.57 (24.92, 26.22)	26.33 (25.07, 27.59)	25.02 (24.38, 25.65)	0.047 *
Sex				
Male	55 (43.3%)	23 (45.1%)	32 (42.1%)	0.739
Female	72 (56.7%)	28 (54.9%)	44 (57.9%)	
Race				0.012 *
White	70 (55.6%)	36 (70.6%)	34 (45.3%)	
Asian	33 (26.2%)	7 (13.7%)	26 (34.7%)	
Other Races	23 (18.3%)	8 (15.7%)	15 (20.0%)	
Desired field of medicine				0.576
Primary care	42 (25.8%)	12 (22.6%)	23 (29.5%)	
Specialty care	77 (47.2%)	27 (50.9%)	33 (42.3%)	
Undecided	44 (27.0%)	14 (26.4%)	22 (28.2%)	
Would like to work with patients under age 18	48 (29.4%)	14 (26.4%)	28 (35.9%)	0.254
Year in medical school				0.685
First Year	35 (21.5%)	10 (18.9%)	20 (25.6%)	
Second Year	73 (44.8%)	25 (47.2%)	34 (43.6%)	
Third Year	27 (16.6%)	9 (17.0%)	9 (11.5%)	
Fourth Year	28 (17.2%)	9 (17.0%)	15 (19.2%)	

* *p* < 0.05; ^1^ CI = Confidence Interval; ^2^ *p*-values from bivariate analyses for examining the association between participants’ perceived health disparity and participants’ characteristics and attitudes.

**Table 2:** Participants’ perceptions related to childhood obesity by their health disparity perception.

Perceptions about	Mean (95% CI ^1^) or Frequency (%)			
	Overall (*n* = 163)	Low Awareness of Health disparity (*n* = 53)	High Awareness of Health Disparity (*n* = 78)	*p*-Value ^2^
**Childhood obesity**				
Reported that it would be likely to encounter childhood obesity in a routine medical visit	133 (95.7%)	49 (94.2%)	74 (96.1%)	0.620
Reported that childhood obesity will affect more of the adolescent population	138 (90.8%)	49 (94.2%)	69 (88.5%)	0.266
Agreed that childhood obesity is a family matter	91 (68.4%)	39 (75.0%)	50 (64.1%)	0.190
Agreed that parents are primarily responsible for their child’s weight	120 (90.2%)	45 (86.5%)	72 (92.3%)	0.283
**Physicians in general**				
Agreed that physicians are not trained to deal with childhood obesity	45 (34.6%)	25 (48.1%)	20 (25.6%)	0.008 *
Reported that physicians can be effective in childhood obesity	90 (70.3%)	34 (66.7%)	56 (72.7%)	0.463
**Participants as future physicians**				
Prepared to treat childhood obesity	60 (47.2%)	29 (56.9%)	31 (40.8%)	0.075
Would be effective in treating childhood obesity as a physician	62 (48.8%)	22 (43.1%)	40 (52.6%)	0.294
**Health Disparity (1–5, Higher score indicating greater agreement with the statements)**				
Low-income American families lack resources to change health behaviors	3.45 (3.29, 3.61)	2.52 (2.37, 2.67)	4.08 (3.97, 4.19)	<0.001 **
Average Americans lack resources to change health behaviors	2.09 (1.92, 2.25)	1.59 (1.38, 1.79)	2.41 (2.19, 2.63)	<0.001 **

* *p* < 0.05; ** *p* < 0.001; ^1^ CI = Confidence Interval; ^2^ *p*-values from bivariate analyses for examining the association between participants’ perceived health disparity and participants’ other perceptions related to childhood obesity.

**Table 3:** Regression model for interaction between health disparity perception and different roles or discussion topics.

Variables	B ^1^ (SE ^2^)		
	Perceived Importance of Roles	Perceived Importance of Discussion Topics (Parents)	Perceived Importance of Discussion Topics (Child Patients)
Intercept	3.76 (0.34) **	4.87 (0.25) **	4.92 (0.31) **
**Roles**			
1 Informal Roles	0.51 (0.25) *	-	-
2 Physicians (reference)	-	-	-
3 Formal Roles	−0.65 (0.29) *	-	-
4 Government	−2.09 (0.45) **	-	-
**Discussion Topics**			
1 Patient Behavior (reference)	-	-	-
2 Access to Safe Environment	-	−0.46 (0.19) *	−0.63 (0.36)
3 School Intervention	-	−1.88 (0.39) **	−1.83 (0.36) **
Health Disparity Perception ^3^	0.06 (0.07)	−0.03 (0.03)	0.004 (0.04)
**Interaction terms**			
Health Disparity Perception x Role 1	−0.003 (0.07)	-	-
Health Disparity Perception x Role 2	-	-	-
Health Disparity Perception x Role 3	0.04 (0.08)	-	-
Health Disparity Perception x Role 4	0.26 (0.13) *	-	-
**Interaction terms**			
Health Disparity Perception x Topic 1	-	-	-
Health Disparity Perception x Topic 2	-	0.06 (0.05)	−0.01 (0.10)
Health Disparity Perception x Topic 3	-	0.27 (0.11) *	0.09 (0.14)

* *p* < 0.05; ** *p* < 0.001; ^1^ B = Unstandardized regression coefficient; ^2^ SE = Standard error; ^3^ Perception that low-income families lack resources to change their health behaviors (score range: 1–5); All regression models were performed after adjusting for participants’ age, race, and perceptions about physicians’ training in childhood obesity.
